# Disentangling vegetation diversity from climate–energy and habitat heterogeneity for explaining animal geographic patterns

**DOI:** 10.1002/ece3.1972

**Published:** 2016-02-09

**Authors:** Borja Jiménez‐Alfaro, Milan Chytrý, Ladislav Mucina, James B. Grace, Marcel Rejmánek

**Affiliations:** ^1^ Department of Botany and ZoologyMasaryk UniversityBrnoCzech Republic; ^2^ Iluka Chair in Vegetation Science and BiogeographySchool of Plant BiologyThe University of Western AustraliaPerthWestern AustraliaAustralia; ^3^ Department of Geography and Environmental StudiesStellenbosch UniversityMatieland 7602StellenboschSouth Africa; ^4^ U.S. Geological Survey700 Cajundome Blvd.LafayetteLouisiana70506; ^5^ Department of Evolution and EcologyUniversity of CaliforniaDavisCalifornia

**Keywords:** Animal diversity, diversity patterns, energy hypothesis, habitat heterogeneity, plant community, productivity, vegetation

## Abstract

Broad‐scale animal diversity patterns have been traditionally explained by hypotheses focused on climate–energy and habitat heterogeneity, without considering the direct influence of vegetation structure and composition. However, integrating these factors when considering plant–animal correlates still poses a major challenge because plant communities are controlled by abiotic factors that may, at the same time, influence animal distributions. By testing whether the number and variation of plant community types in Europe explain country‐level diversity in six animal groups, we propose a conceptual framework in which vegetation diversity represents a bridge between abiotic factors and animal diversity. We show that vegetation diversity explains variation in animal richness not accounted for by altitudinal range or potential evapotranspiration, being the best predictor for butterflies, beetles, and amphibians. Moreover, the dissimilarity of plant community types explains the highest proportion of variation in animal assemblages across the studied regions, an effect that outperforms the effect of climate and their shared contribution with pure spatial variation. Our results at the country level suggest that vegetation diversity, as estimated from broad‐scale classifications of plant communities, may contribute to our understanding of animal richness and may be disentangled, at least to a degree, from climate–energy and abiotic habitat heterogeneity.

## Introduction

One of the main aims of biogeography and ecology is to understand spatial diversity patterns and their major determinants. From a plethora of hypotheses focused on explaining geographic variation in species diversity, those related to climate–energy and habitat heterogeneity have received major empirical support (Currie et al. 2004; Turner and Hawkins 2004). The *climate–energy hypothesis* roots in the concept of productivity, proposing that the availability of water and energy controls plant productivity, which in turn has an influence on the diversity of herbivores and associated carnivores through bottom‐up forcing (Turner and Hawkins 2004). A complement to this view is the *ambient‐energy hypothesis* that states that climatic factors may also directly influence the physiology of animals, especially endotherms (Currie 1991; Hawkins et al. 2003). In addition, habitat (environmental) heterogeneity has been proposed as an important driver of species diversity, with similar or higher predictive power than climate and energy (Kerr and Packer 1997; Stein et al. 2014). In its simple form, *the habitat heterogeneity hypothesis* posits that the spatial variation of abiotic or biotic factors shapes the realized niches of plants and animals in a given territory (Kerr and Packer 1997; Stein et al. 2014).

The impacts of climate–energy and habitat heterogeneity on animal diversity are obviously linked to plant diversity, as stated by Hutchinson (1959): “The extraordinary diversity of terrestrial fauna is clearly due largely to the diversity provided by terrestrial plants.” This relationship has been extensively tested, and a meta‐analysis by Castagneyrol and Jactel (2012) provided strong support for the use of plant species richness as a predictor of animal diversity, emphasizing the importance of cross‐taxon correlates for understanding biodiversity patterns. However, the role of plants in determining patterns of animal diversity might also be linked to the attributes of plant communities in nature. Plant community processes, such as environmental filtering, interspecific interactions, dispersal limitation, biogeographic history, and neutral processes (Vellend 2010), are all to a large extent influenced by plant–animal interactions, including herbivory, pollination, and seed dispersal. Therefore, the diversity of plant community types (defined at any level of organization in a geographic area) is expected to correlate with animal diversity by reflecting different attributes of vegetation in ecosystems (Hooper and Vitousek 1997; Stein et al. 2014). This view was introduced as the *vegetation structure hypothesis*, stating that the vegetation physiognomy may shape the availability of niches for animals (MacArthur and MacArthur 1961), and later expanded by studies arguing for a stronger influence of *vegetation composition* or *floristics* (Rotenberry 1985), opening an unresolved debate about the relationship between vegetation and animal diversity.

**Figure 1:**
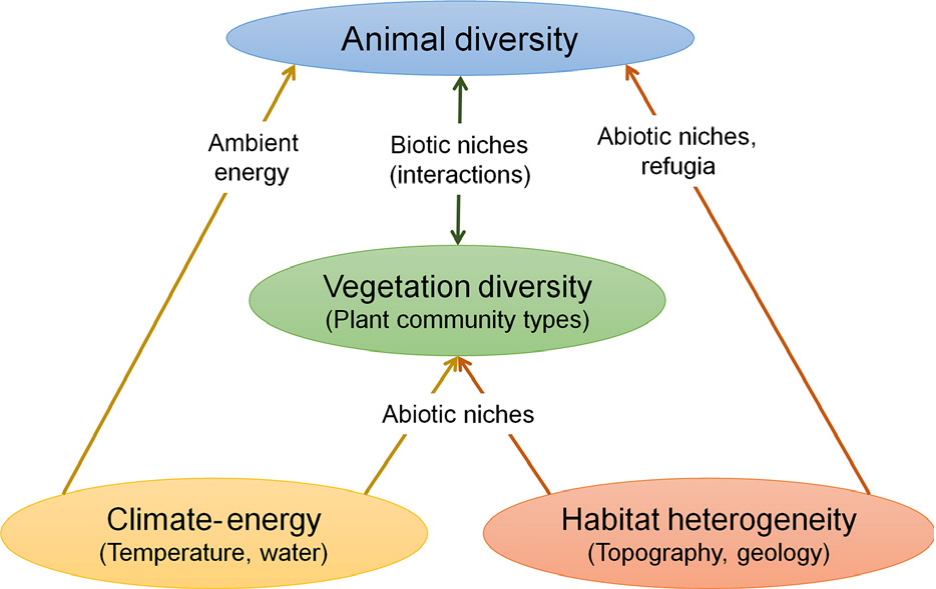
A conceptual framework with the assumed influence of climate–energy, habitat heterogeneity, and vegetation diversity for explaining animal geographic patterns.

The complexity of plant–animal relationships creates a conceptual difficulty since it is far from trivial to disentangle the role of plant communities as a causal driver of animal diversity or as a coexisting counterpart controlled by broad‐scale abiotic factors. Although plants and animals alike are influenced by spatial and historical factors (Field et al. 2009), plant communities are at the same time a source of food and shelter for the latter (Castagneyrol and Jactel 2012), thereby affecting animal richness. However, we do not know of any rigorous tests looking at the conceptual integration of vegetation diversity (i.e., structure and composition of plant communities), climate–energy, and habitat heterogeneity hypotheses. Here, we propose a conceptual framework by which vegetation diversity (including both structure and composition) represents a necessary bridge between abiotic factors and animal richness (Fig. 1). According to this hypothesis, plant populations respond to abiotic factors, forming plant communities that vary in functional characteristics such as productivity and functional diversity (at this point, we intentionally disregard the important role of soil biota for the sake of simplification). The structure and floristic complexity of the plant communities provide biotic niches for animals, including bidirectional plant–animal interactions. In addition, animals may also be directly influenced by climate (as suggested by the ambient‐energy hypothesis) and the abiotic habitat heterogeneity (through abiotic niches). This conceptual framework integrates the general expectations of both the climate–energy and the habitat heterogeneity hypotheses (but it contrasts with the current trend that considers vegetation diversity as a surrogate of habitat heterogeneity: Jetz and Rahbek 2002; Qian 2007; Keil et al. 2012). Thus, we presume that biotic effects of vegetation result from not only the structure (physiognomy) but also the composition of plant communities (as predicted from previous studies at different scales, Rotenberry 1985; Fleishman et al. 2003).

In this paper, we attempt to disentangle the effect of vegetation diversity from the effects of climate–energy and abiotic habitat heterogeneity as explanations of animal geographic patterns. In our investigation, we analyze regional drivers of animal diversity in four vertebrate (mammals, birds, amphibians, and reptiles) and two invertebrate (beetles and butterflies) groups across large European regions. We considered species richness (regional number of species), the most common estimate of diversity, and regional dissimilarity (variation, or turnover in species composition) to identify spatial diversity patterns at broad scales (Roy et al. 2004). We expected that, at least for certain animal groups tightly dependent on plant communities (e.g., those with short‐distance dispersal and narrower ecological niches), predictors of vegetation diversity might account for some variation not explained by factors related to climate–energy and abiotic habitat heterogeneity. We also expected that vegetation–animal relationships at the regional scale might change across different animal groups and across the two facets of diversity (richness and dissimilarity).

## Methods

### Animal richness data

We used regional species lists for six well‐studied animal groups in Europe. In total, we worked with data from 20 regions, most of them corresponding with European countries (hereafter called “countries”) for which we managed to compile complete data for animal diversity and its potential predictors (Fig. S1). The data on mammals were extracted from the *Societas Europaea Mammalogica* as compiled by Heikinheimo et al. (2007), consisting of presence/absence records for 146 species in 50 km × 50 km grids. Data on birds were collected from the official census of European birds (BirdLife International/European Bird Census Council 2000), reporting accurate presence records for 253 bird species at the country level. Data for amphibians and reptiles were obtained from the *European Herps by Country* website (http://www.cyberlizard.plus.com/, accessed April 2014) that was subsequently collated using the new atlas of amphibians and reptiles in Europe (http://na2re.ismai.pt/atlas.php).

We also used data on distribution of 2890 European carabid beetles (Carabidae) from www.carabids.org, derived mainly from the *Catalogue of Palaearctic Coleoptera* (Löbl and Smetana 2003). Finally, we compiled data on 4005 species of butterflies from a pan‐European revision by Karsholt and Razowoski (1996), excluding small moths (microlepidoptera) since these are in general poorly studied and their distribution data may be less accurate. Data for the six animal groups were converted to species × country matrices and total species richness values were estimated for each country (Fig. S1). The completeness of these country checklists is expected to be high given the large spatial scale and the effort invested in compilation of the original data sources.

### Predictors

As a surrogate for available atmospheric energy, we focused on potential evapotranspiration (PET, mm/year), which has been recognized as one of the best predictors of species richness in many animal groups (Currie 1991; Hawkins et al. 2003; Turner and Hawkins 2004). For our purpose, PET is preferable to actual evapotranspiration because the latter is more related to water availability, plant productivity, and vegetation composition (Fisher et al. 2011). Mean annual PET was obtained from the Global‐PET Database (www.cgiar-csi.org), which uses the temperature radiation equation of Hargreaves (1994). This procedure provides a good agreement with independent estimates of PET, and it has been recommended for broad‐scale studies (Zomer et al. 2006). We also extracted mean annual precipitation (MAP, mm/year) from the WorldClim database (www.worldclim.org) as a complementary variable to account for water availability. For the sake of simplicity, we excluded other WorldClim variables that were correlated with PET, such as mean annual temperature (Pearson *r* = 0.84, *P* < 0.001) and temperature sum for the growing season (*r* = 0.85, *P* < 0.001).

To measure abiotic habitat heterogeneity, we calculated the range of altitude (ALTr, in meters) as the difference between minimum and maximum values per country. This is among the most informative predictors reflecting abiotic habitat heterogeneity at broad scales, and it is mainly used as a surrogate for topographic diversity (Veech and Crist 2007). We also tested other variables reflecting topographic diversity within regions, in particular standard deviation, roughness index (as proposed by Stein et al. 2015), and Shannon index per country; however, they were found to be highly correlated with ALTr (*r* > 0.80) and hence further discarded for the sake of clarity. In addition, we quantified the geological diversity of each country by using the geological raster map of the European Soil Survey (Panagos et al. 2012). We extracted the number of substrates per country using the classification of parent material at the third level of the survey that reflects the diversity of major bedrock categories (min = 7, max = 43).

Vegetation diversity (VEG) was calculated from a database of plant community types compiled for European countries (Jiménez‐Alfaro et al. 2014). We focused on the hierarchical level of “alliances,” which represent groups of plant associations with similar composition, physiognomy, and habitat requirements (Peet and Roberts 2013), and are useful for the classification of vegetation at (sub)continental spatial scales. In the European context, the alliances are mainly based on floristic composition corroborating (at a large extent) the conceptual basis of alliances as used in the North American vegetation classification approach (Jennings et al. 2009). We created a presence/absence matrix featuring a total of 746 alliances representing the vegetation types reported from European countries. The number of alliances per country ranged from 88 (the Netherlands) to 331 (Spain). Data for each country and information on the respective sources are provided in Jiménez‐Alfaro et al. (2014). The spatial data were handled using ArcGIS 10.2 (ESRI, Redlands, CA).

### Correlates of animal diversity

We calculated pairwise correlations between animal diversity and the main predictors reflecting the number of vegetation types (VEG), abiotic habitat heterogeneity (ALTr), and climate–energy (PET). Spearman's rank correlation *ρ* was used to measure, for each animal group, statistical relationships between the number of species per country and the predictors. The correlations with compositional patterns were tested for the same predictors using Mantel tests. This method is appropriate to model pairwise dissimilarities as a function of pairwise environmental variables and it is well adapted for analyzing only one gradient at a time (Legendre and Fortin 2010). Jaccard similarity coefficient was selected as an appropriate measure of resemblance in animal species composition accounting for presence/absence data excluding joint absences, assuming that two samples (countries) missing a given species are not necessarily similar (Anderson et al. 2011). Resemblance for VEG was also calculated using the Jaccard coefficient, whereas for the quantitative variables ALTr and PET, we used Euclidean distance as a measure of similarity. The Mantel tests were computed for each of the predictors separately (i.e., simple Mantel tests) using PAST (Hammer et al. 2001) and applying a Monte Carlo test with 5000 permutations.

### Models for species richness

We first created GLMs (generalized linear models) to find the predictors that best explained the species richness of the six animal groups, using a Poisson distribution and log‐link function in R (version 2.15.3; R Core Team, Vienna, AT). Since our sample size was relatively small (*N* = 20) and we expected colinearity between predictors, we created a first model with VEG, ALTr, and PET only, selecting the best predictors by a stepwise forward procedure using the AIC (Akaike's information criterion). Only the selected variables with a significant contribution to the final model (*P* < 0.05), as verified in ANOVA type II test, were finally considered. We repeated the process including those variables together with new ones to control for the effect of country size (AREA), geological diversity (GEOL), and precipitation (PREC), because they might be potentially important for explaining animal richness (Jetz and Fine 2012; Homburg et al. 2013). We also assessed spatial autocorrelation that could influence our results, and analyzed model residuals using the Moran's *I*. The tests showed lack of spatial autocorrelation, likely due to the high variation among countries as previously stated at similar spatial resolution (Jetz and Fine 2012), and thus, the spatial autocorrelation was not considered in the models.

Variance partitioning was used to compare the relative importance of VEG, ALTr, and PET. This procedure allowed us to discriminate the pure effects of the three predictors and the shared variation, and therefore provided a better understanding of their proportional influence on animal diversity patterns. In order to estimate the explained variation in the GLMs, we first calculated pseudo‐*R* ^2^ values according to the McFadden's formula using the “pR2” function in R package *pscl* (Jackman 2012). Variance partitioning was then computed for each model using the pseudo‐*R* ^2^ values with the function “varPart” in package *modEvA*. We calculated the proportion of explained deviance for VEG, PET, ALTr, and their paired combinations. Since this approach does not quantify unexplained variation, we calculated the proportions of explained deviance for each of the factors included in the GLMs.

As a complement to the GLMs, we used structural equation modeling (SEM; Grace et al. 2012, 2015) to consider the general hypothesis that animal diversity has distinct responses to climate/energy, heterogeneity, and vegetation diversity. Rather than evaluating separate models for each animal group, we took advantage of the high degree of correlation among animal diversity patterns to construct a latent variable model representing animal diversity as a general response. Because of the nature of the data sample, we decided to adopt a Bayesian approach to the SEM (Grace et al. 2012), focusing on estimating the strengths of various direct and indirect pathways related to the overall hypothesis. For practical reasons, we chose to ignore feedback effects of animal diversity on plant diversity, as (1) we lack variables that would unambiguously identify a feedback effect; and (2) the focus of our study was primarily on understanding drivers of animal diversity. For our analyses, we used the Amos software package (IBM 2014; version 22) and employed Markov chain Monte Carlo methods with neutral priors. Multiple runs were used to ensure consistent estimates. Because our sample is a nearly complete representation of the study area, we focused on estimation of effect magnitudes rather than hypothesis testing (as uncertainty goes to zero in complete samples).

### Models for species composition

We created multiterm models for explaining variation in animal species composition using redundancy analysis (RDA) by testing the null hypothesis that species composition and its spatial distribution were independent of a given independent variable. The best predictors were chosen by a forward selection procedure adding new variables according to their decreasing eigenvalues until they were nonsignificant (*P* > 0.05) by using CANOCO 5.0 (www.canoco5.com). Given VEG is a multivariate variable reflecting compositional patterns across countries, we reduced its complexity to the main principal components (VEG‐pc1, VEG‐pc2, VEG‐pc3, and VEG‐pc4), which together accounted for 78% of the variation. These variables were included in the RDAs together with ALTr, GEOL, PET, and PREC. Since our main aim was to compare the predictors related to energy–climate, habitat heterogeneity, and vegetation, the influence of spatial variation was not tested here but in the following procedure.

We used adjusted‐*R* ^2^ (Peres‐Neto et al. 2006) to estimate the explained variation in the RDAs and adopted a stepwise selection procedure in building a model with significant variables within each data set with a permutation *P*‐value of 0.05. The total sum of RDA eigenvalues was used to calculate the variance partitioning (Borcard et al. 1992) and the proportion of explained variance for each predictor. The other variables (PREC, GEOL) produced nonsignificant results in the RDAs and were therefore not included in the models. The variable VEG was decomposed into the PCA axes as explained above. Since the variation in animal species composition can be strongly related to pure spatial patterns as an effect of biogeographic history, we also compared the proportion of the explained variance of the three predictors with the spatial structure of the data. We computed a set of multiscale spatial variables using eigenvectors of principal coordinates of neighbor matrices, PCNM (Borcard and Legendre 2002), assuming that the variables related to broad scales (associated with first and large eigenvalues) had the greatest explanatory power. To assess the spatial variation accounted for by the three main predictors analyzed before, we computed separate RDAs with variance partitioning between PCNM *n* axes and (1) the main vegetation axes (VEG‐pc1‐4); (2) potential evapotranspiration (PET); and (3) altitudinal range (ALTr). Pure and shared effects were obtained for the three predictors and for the six animal groups.

## Results

Mantel tests showed significant relationships between the species richness of all the animal taxa, vegetation diversity (VEG), and altitudinal range (ALTr). For these two predictors, the highest correlations were detected for amphibians, beetles, and butterflies, followed by mammals (Table 1). Potential evapotranspiration (PET) was significantly related only to the species richness of reptiles, but in this case showed a very high correlation. The results of the Mantel tests also showed significant correlations between faunal compositional similarities and vegetation similarities, with the highest correlation found for amphibians, beetles, and butterflies, followed by mammals, reptiles, and birds. PET showed significant relationships but with consistently lower values for all animal groups, while ALTr was not correlated with any animal group.

**Table 1:** Correlations between animal species richness (Spearman's rank correlation *ρ*), animal species composition (Mantel *R* ^2^), and the predictors reflecting vegetation diversity (VEG), altitudinal range (ALTr), and potential evapotranspiration (PET). ns: not significant

	VEG		ALTr		PET	
Species richness	*ρ*	*P*‐value	*ρ*	*P*‐value	*ρ*	*P*‐value
Mammals	0.66	0.002	0.60	0.005	0.30	0.219^ns^
Birds	0.45	0.045	0.60	0.005	0.40	0.082^ns^
Reptiles	0.53	0.015	0.52	0.019	0.82	<0.001
Amphibians	0.79	<0.001	0.64	0.002	0.55	0.132^ns^
Beetles	0.70	<0.001	0.69	0.001	0.30	0.193^ns^
Butterflies	0.79	<0.001	0.77	0.001	0.34	0.139^ns^

Species composition	*R* ^2^	*P*‐value	*R* ^2^	*P*‐value	*R* ^2^	*P*‐value
Mammals	0.73	0.002	0.13	0.150^ns^	0.42	<0.001
Birds	0.54	0.002	0.13	0.148^ns^	0.42	<0.001
Reptiles	0.67	0.002	0.13	0.151^ns^	0.42	<0.001
Amphibians	0.78	0.002	0.13	0.156^ns^	0.42	<0.001
Beetles	0.89	0.002	0.13	0.148^ns^	0.42	<0.001
Butterflies	0.87	0.002	0.13	0.145^ns^	0.41	<0.001

The results of GLMs identified VEG (number of vegetation types) as the best predictor of species richness in amphibians, beetles, and butterflies (Table 2). For amphibians, VEG was the unique selected variable (accounting for 80% of the explained deviance), while the species richness of beetles and butterflies was also explained by up to four variables including AREA and ALTr. In contrast, the species richness of mammals and birds was explained by different models, with ALTr and PREC as the first and second predictors, accounting a total of 58% of explained deviance in the two animal groups. Finally, the species richness of reptiles showed a very different response, with PET as the main predictor, followed by ALTr and contributing a total of 80% of explained deviance.

**Table 2:** Summary of multiple‐term GLMs and the variables selected after forward selection for explaining animal species richness in 20 European countries. First selected predictors are in bold. “Explained” indicates the % of explained deviance

	Variable	*Z*‐value	Explained (%)	*P*‐value
Mammals	**ALTr**	**4.75**	**40**	**<0.001**
	PREC	**−**3.15	18	0.001
Birds	**ALTr**	**5.11**	**33**	**<0.001**
	PREC	**−**3.67	25	<0.001
Reptiles	**PET**	**10.27**	**69**	**<0.001**
	ALTr	4.85	11	<0.001
Amphibians	**VEG**	**6.13**	**80**	**<0.001**
Beetles	**VEG**	**9.74**	**65**	**<0.001**
	AREA	12.74	12	<0.001
	ALTr	12.04	4	<0.001
	PREC	**−**8.02	3	<0.001
	GEOL	**−**7.91	2	<0.001
Butterflies	**VEG**	**17.49**	**69**	**<0.001**
	ALTr	12.16	7	<0.001
	PREC	**−**14.01	4	<0.001
	AREA	**−**11.20	3	<0.001
	PET	**−**7.87	3	<0.001

**Figure 2:**
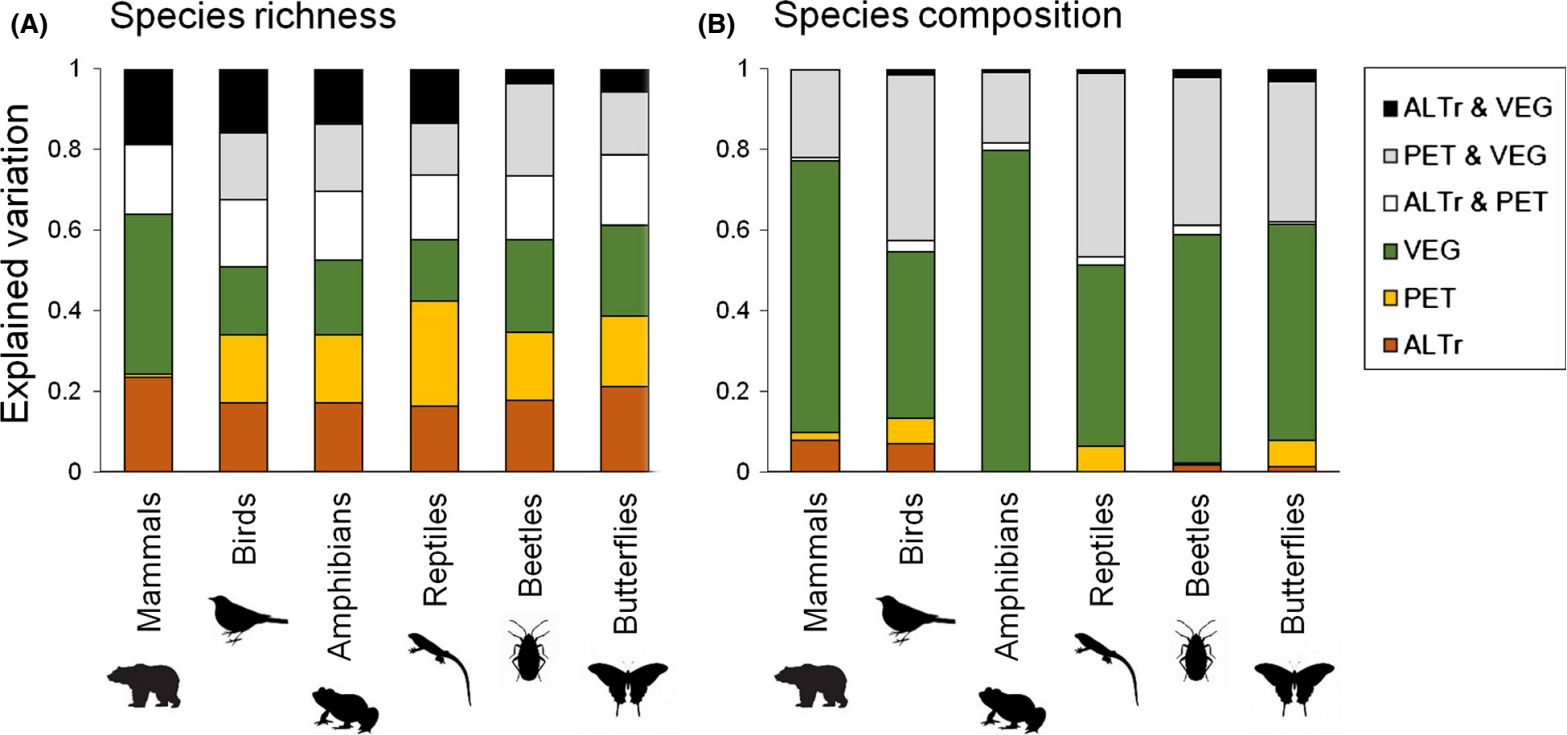
Variation partitioning for the influence of VEG (vegetation), altitudinal range, and potential evapotranspiration when these predictors are modeled to explain diversity patterns of six animal groups across Europe. Explained variation reflects the % of deviance from generalized linear models with pseudo‐*R* ^2^ for species richness, and the % of variance from redundancy analyses with adjusted‐*R* ^2^ for species composition (in this case, VEG summarizes the first four axes of a principal component analysis computed for the variation of plant community types).

According to variance partitioning (Fig. 2A), VEG particularly contributed to mammal richness and provided the highest contributions to richness in amphibians, beetles, and butterflies. ALTr was the second most important contributor to the richness of mammals, and both ALTr and VEG exhibited similar effects on birds, amphibians, beetles, and butterflies. The fact that we detected many significant shared contributions of ALTr & PET or PET & VEG explains why PET was excluded in most of the GLMs. The combination of ALTr & VEG also explained a notable portion of the variation, with a decreasing magnitude from mammals to butterflies.

**Figure 3:**
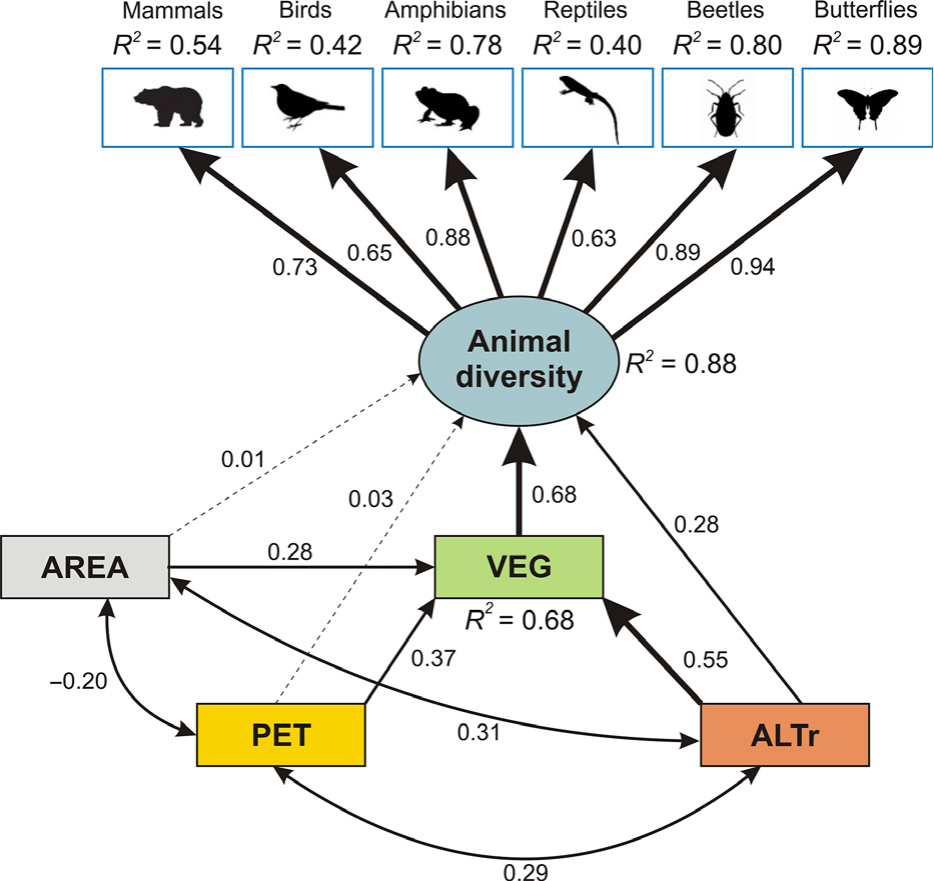
Results for the structural equation model. Animal diversity was represented as a latent variable (in an oval), while the boxes represent observed variables. The effect of sample area was included as a control variable. Arrow widths reflect the standardized path coefficients whose precise values are indicated by accompanying numbers.

The structural equation model showed generally similar results to the GLMs, with VEG and ALTr having the strongest relationships to animal diversity and also to the different animal groups (Table 3). Nevertheless, VEG was the predictor with the highest direct contribution to animal diversity within the model (Fig. 3). While PET and AREA did not provide relevant relationships with animal diversity, they contributed indirectly to the variation of VEG. There was also an important (indirect) relationship between ALTr and VEG (0.55), but in this case ALTr also contributed to animal diversity directly (0.28). Overall, VEG was the variable with the highest correlations with other predictors and with the highest contribution to animal diversity and to the animal groups separately.

**Table 3:** Standardized effects obtained by structural equation modeling for explaining European animal diversity as a whole and for six animal groups, using potential evapotranspiration (PET), altitudinal range (ALTr), vegetation diversity (VEG), and area (AREA) as explanatory variables. Direct, indirect, and total effects reflect path strengths within the model. Coefficients between 0.25 and 0.50 are considered to be moderately strong, and those >0.5 are considered strong

	PET	ALTr	VEG	AREA
*Total animal diversity*				
Total effect	0.25	0.65	0.69	0.19
Direct effect	0.00	0.28	0.69	0.00
Indirect effect	0.25	0.37	0.00	0.19
*Animal groups (total effects)*				
Butterflies	0.24	0.61	0.65	0.18
Beetles	0.23	0.57	0.62	0.17
Reptiles	0.17	0.41	0.44	0.12
Amphibians	0.23	0.57	0.61	0.17
Birds	0.17	0.42	0.45	0.12
Mammals	0.19	0.47	0.50	0.14

In concordance with the relationships detected by the Mantel tests, RDAs selected VEG‐pc and PET as the main predictors of species composition for the six animal groups (Table 4). The PCA axes of VEG were the unique selected variables for mammals (total variation explained: 56.4%) and amphibians (50.8%). In the other four taxa, PET was selected as the best predictor, but consistently followed by the PCA axes of VEG, altogether contributing to an explained variance total of 52.8% in birds, 55.8% in reptiles, 47.2% in beetles, and 58.7% in butterflies (in this case with an additional effect of ALTr).

**Table 4:** Summary of multiterm RDAs and the variables selected after forward selection for explaining animal species composition across 20 European countries. The first selected predictors are in bold. “Explained” indicates the % of explained variation (adjusted‐*R* ^*2*^ × 100). VEG‐pc1 through VEG‐pc4 stand for the four main axes of a PCA performed with the compositional variation of vegetation types across the study regions

	Variable	Pseudo‐*F*	Explained (%)	*P*‐values
Mammals	VEG‐pc2	3.9	18.0	0.002
	VEG‐pc1	4.4	16.8	0.002
	VEG‐pc3	3.8	12.8	0.002
	VEG‐pc4	3.0	8.8	0.002
Birds	PET	5.7	24.2	0.001
	VEG‐pc1	3.6	13.1	0.001
	VEG‐pc2	2.3	7.9	0.001
	VEG‐pc3	2.4	7.6	0.001
Reptiles	PET	5.5	23.3	0.002
	VEG‐pc1	3.8	14.1	0.002
	VEG‐pc3	4.0	12.5	0.002
	VEG‐pc4	2.0	5.9	0.006
Amphibians	VEG‐pc1	4.9	21.4	0.002
	VEG‐pc3	3.9	14.6	0.002
	VEG‐pc2	2.2	7.8	0.002
	VEG‐pc4	2.1	7.0	0.008
Beetles	PET	3.9	17.7	0.001
	VEG‐pc1	3.2	13.0	0.001
	VEG‐pc3	2.6	9.6	0.001
	VEG‐pc4	2.0	6.9	0.001
Butterflies	PET	4.7	20.7	0.002
	VEG‐pc1	3.6	13.9	0.002
	VEG‐pc3	3.2	10.9	0.002
	ALTr	2.6	13.2	0.002

**Figure 4:**
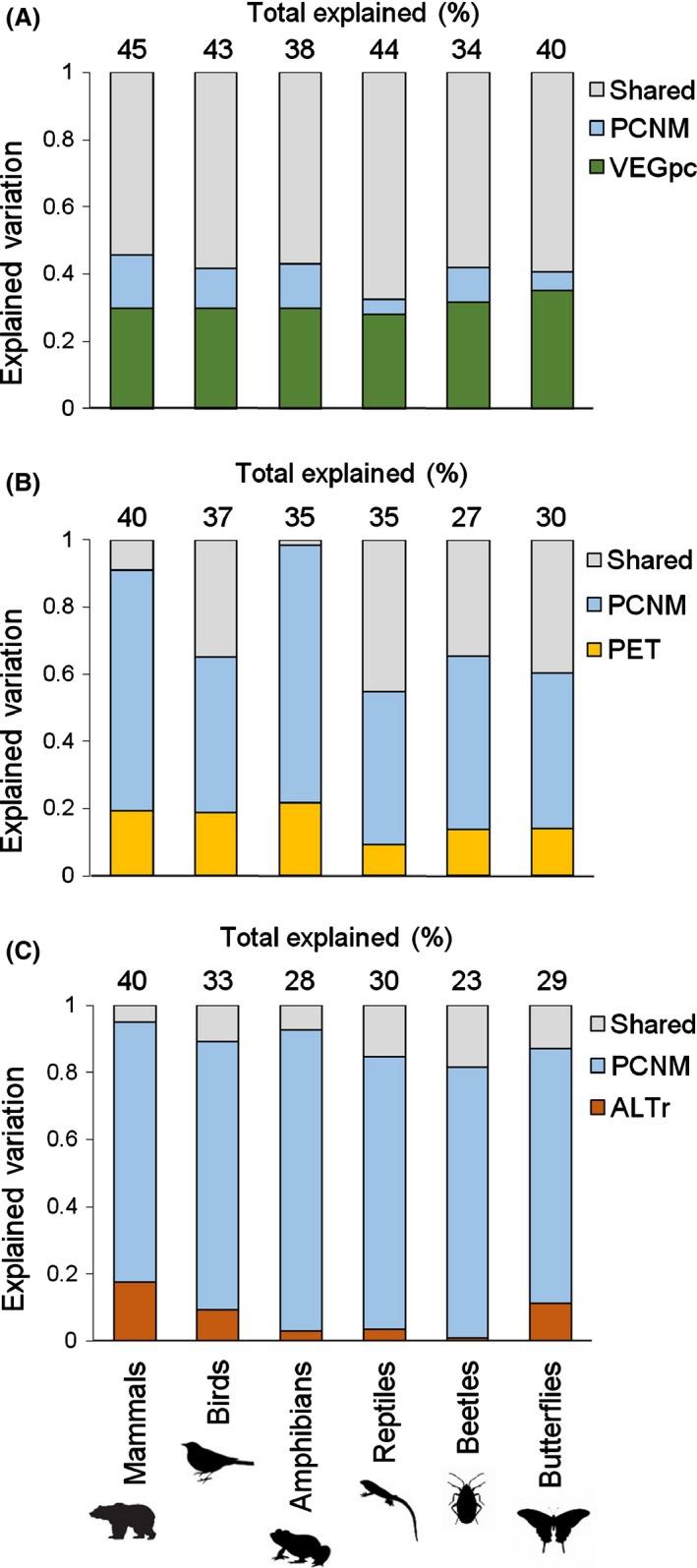
Variation partitioning between the spatial structures reflected by principal components of neighbor matrices (PCNM) and the predictors used for explaining animal species composition across Europe: (A) VEG‐pc, summarizing the first four axes of a PCA; (B) PET, potential evapotranspiration; and (C) ALTr, altitudinal range.

The variance partitioning of species composition (Fig. 2B) showed that the influence of the PCA axes of VEG dominated the proportion of explained variance in all cases. A remarkable point is that the effects of PREC and GEOL were not significant in any case, and therefore, the variance partitioning for different taxa was more related to the RDAs than in the GLMs. However, the patterns of variable contributions were clearly different, as reflected by the shared proportion of PET & VEG that was much higher than the other two combinations. When compared with the spatial structure estimated by the PCNM axes, the shared contribution was much higher than when VEG (Fig. 4A), PET (Fig. 4B), or ALTr (Fig. 4C) were used. In addition, VEG contributed a relatively high proportion of explained variance that was not accounted for by the pure or shared spatial effects. In contrast, PET and especially ALTr contributed very little to the explained variance in the composition of most animal groups, since the influence of the pure spatial effect appeared to be much more relevant.

## Discussion

### Vegetation as a predictor of animal diversity

Our study shows strong correlations between broad‐scale patterns of vegetation and animal diversity in the six animal groups tested. More importantly, in several groups we found a larger contribution of plant community types (VEG) than altitudinal range (ALTr), potential evapotranspiration (PET), and area (AREA) that were consistently detected by pairwise correlations, GLMs, and SEMs. These results support the idea that vegetation diversity, estimated via the structure and composition of plant community types, may provide unique correlates with broad geographic animal patterns not accounted for by climate and abiotic habitat heterogeneity. While some studies have shown the importance of testing vegetation–animal relationships (Steinmann et al., 2011; Fløjgaard et al., 2011), to our knowledge broad‐scale correlates between plant community types and animal diversity have not been tested before.

In agreement with our expectations, the discerned contribution of vegetation diversity varied across animal groups and facets of diversity. The contribution of VEG was especially important in amphibians, beetles, and butterflies, which have smaller home ranges and lower spatial mobility than mammals and birds. The significant effect of VEG on amphibians (reflected in GLMs and SEMs) is in contrast to other studies at similar scales (Qian 2010; Poessel et al. 2013) that have suggested climate–energy (via precipitation, water availability, or PET) as the main driver of amphibian diversity; nonetheless, analogous estimates of vegetation diversity have not been previously evaluated. Similarly, the patterns of richness in beetles and butterflies were mainly explained by VEG and to a lesser extent by PET and ALTr. These results contrast with other studies that found higher support for abiotic habitat heterogeneity at broad scales (Tews et al. 2004). However, our results agree with fine‐scale studies suggesting that vegetation diversity is the main driver of species richness in invertebrates (Jonsson et al. 2009).

Variance partitioning of animal richness also revealed that the contribution of VEG is relevant as an independent factor, showing different magnitudes of shared explained variation with PET and ALTr across the six animal groups. These results were supported by the structural equation model, reflecting the strongest direct contribution of VEG to the entire animal richness, and the highest coefficients provided by VEG and ALTr for the six animal groups. Beetles and butterflies, but also amphibians, were more tightly related to vegetation cover than birds and animals, which frequently migrate over regions and habitat types, explaining the high contribution of VEG in those groups. Birds are more mobile than mammals, and therefore, they are expected to be less dependent on the variation of vegetation diversity and more influenced by the direct effect of abiotic variables. Although the value of vegetation diversity for explaining species richness in mammals and birds was substantially lower, this effect is not inconsequential since it still provides a unique contribution. Contrary to other studies (Barton et al. 2014), the relatively high contribution of VEG to mammals likely resulted from the higher resolution of our data in terms of plant community types, likely reflecting variation in vegetation (floristic) composition. In addition, relatively low correlations of VEG with respect to avian diversity may result from insufficient information about vertical structure of vegetation at the scale of study, given that the importance of this factor has been mainly recognized at finer scales (MacArthur and MacArthur 1961; Barton et al. 2014). Finally, the high predictive power of potential evapotranspiration in reptile richness is not surprising, as the importance of energy and climate (e.g., temperature) for the diversity of this ectothermic group is well known (Poessel et al. 2013).

Our results also provide insights into vegetation–animal correlates in regional similarity. In contrast with other studies exploring beta‐diversity in terms of within‐regional variation (Veech and Crist 2007), we provide a correlative approach to assess between‐region variation of animal species composition. Testing compositional variation is in many cases necessary to complement species richness for a better understanding of geographic patterns (Roy et al. 2004), but this simple approach has hardly been used for testing vegetation–animal relationships at broad scales. In agreement with the patterns of species richness, we found a strong influence of VEG for explaining animal between‐region compositional variation, followed by a moderate explanatory value of PET and a poor effect of ALTr. Again, the strongest correlations between animal and vegetation variation were detected for amphibians, beetles, and butterflies. These results support that at the scale of our study, the patterns of vegetation diversity are highly correlated with the patterns of animal diversity, as might be expected from a common biogeographic history (Storch et al. 2003; Heikinheimo et al. 2012).

Although the relatively low and uniform performance of ALTr and PET could be expected given the simplicity of these estimates in contrast with the high variation of plant community types, the results of the variance partitioning provided two interesting outputs. First, VEG was partially correlated with the variation of PET (Fig. 2) reflecting the correlation between climatic variation and vegetation variation across the study area. Second, the contribution of VEG and especially PET was intrinsically related to the spatial variation among countries (Fig. 3), resulting in difficulty disentangling the spatial effect in most of the explained variation. This reflects that the variation in vegetation, climate, and animal diversity across the studied regions has a similar biogeographic component, as may be expected for the spatial complexity of Europe. However, the unique contribution of VEG that was not linked to any other environmental or spatial factor was the highest among the tested predictors, suggesting closer relationships between vegetation and animal diversity.

### Scale effect and further perspectives

Our study provides evidence that vegetation diversity, when estimated from the variation of plant community types, can be used as an explanatory variable with independent effect on broad‐scale animal richness. Although we focus on a relatively low number of regions, the high quality of the data and the current knowledge on biodiversity in Europe support the reliability of the observed patterns. However, we realize that the scale of region–country is too broad for interpreting the results in terms of causal relationships between plant communities and animals. Patterns of biodiversity may vary with spatial scale (Crawley and Harral 2001) and vegetation–animal correlates can change from broad to local scales. Thus, our study mainly supports the idea that, at broad scale and low spatial resolution, vegetation diversity can be disentangled from abiotic components to explain animal diversity. This contrasts with the general assumption that vegetation diversity is poorly differentiated from abiotic drivers and especially from abiotic habitat heterogeneity (Stein et al. 2014).

Although vegetation diversity is to some extent correlated with abiotic factors (as shown in Fig. 2), we found that it outperforms them in explaining geographic patterns of animal taxa that are more dependent on local plant communities, given their dispersal abilities and ecological specialization. This view is largely congruent with the biodiversity hypotheses claiming that, at broad spatial scales and in terrestrial habitats, climate and productivity play the most important role in determining animal species richness (Field et al. 2009). Our data are nonetheless limited to infer the relationships among vegetation diversity, productivity, and the organization of different trophic levels of animal diversity (Huston 1999), and more studies at higher spatial scales are still needed to understand the underlying mechanisms in the observed patterns.

Overall, this study provides an attempt to incorporate vegetation diversity in broad‐scale studies dealing with animal geographic patterns, assuming the role of plant communities as the main source of primary productivity. At broad scales, vegetation diversity is generally recognized to respond to climatic factors (e.g., evapotranspiration, Fisher et al. 2011) and also to abiotic habitat heterogeneity (e.g., altitudinal range, Jiménez‐Alfaro et al. 2014). However, the effect of these drivers on plant diversity is generally estimated in terms of plant species richness (Castagneyrol and Jactel 2012). Our results clearly suggest that vegetation diversity, interpreted in terms of compositional diversity of plant communities (Rotenberry 1985), can provide a meaningful interpretation of animal geographic patterns. This validates at least partially the framework proposed in Figure 1, supporting usefulness of vegetation classification integrating both species composition and physiognomy, an important element missing in broad‐scale studies exploring patterns of animal diversity (Jetz et al. 2009).

We are aware that detailed information about plant community types may be difficult to get in many regions, and that even so, models for explaining animal diversity may vary widely when applied to regions with different environments or history (Andrews and O'Brien 2000; Qian 2010; Svenning et al. 2011). However, the increasing availability of massive biodiversity data and the importance of understanding plant–animal correlates for conservation assessment justify a search for universal drivers of species diversity (Stein et al. 2014). Similar studies are much needed for assessing continental and global biodiversity patterns, and for testing whether vegetation diversity is expected to provide strong predictors of animal diversity at different spatial scales. Until now, the lack of such studies is probably due to the low accessibility of vegetation data, but this scenario is changing quickly since large databases are now providing new information about diversity gradients (Lamanna et al. 2014). Thus, we encourage ecologists to integrate plant community data into biodiversity models and to apply the proposed framework as a starting point to approach models at different spatial scales and in different contexts of environmental drivers.

## Conflict of Interest

None declared.

## Supporting information


**Figure S1**. Total species richness collected in 20 European regions analysed for testing the influence of surrogates of climate‐energy, habitat heterogeneity and vegetation diversity on six animal groups.
