# Supplementary material for: Disentangling vegetation diversity from climate–energy and habitat heterogeneity for explaining animal geographic patterns
Source: Ecol Evol. 2016 Feb 9;6(5):1515–26. doi: 10.1002/ece3.1972 (PMC4747316; doi:10.1002/ece3.1972)

Figure S1. Total species richness collected in 20 European regions analysed for testing the influence of surrogates of climate-energy, habitat heterogeneity and vegetation diversity on six animal groups. Nordic countries were merged into a unique region (Fennoscandia). Colours reflect 5 richness classes.

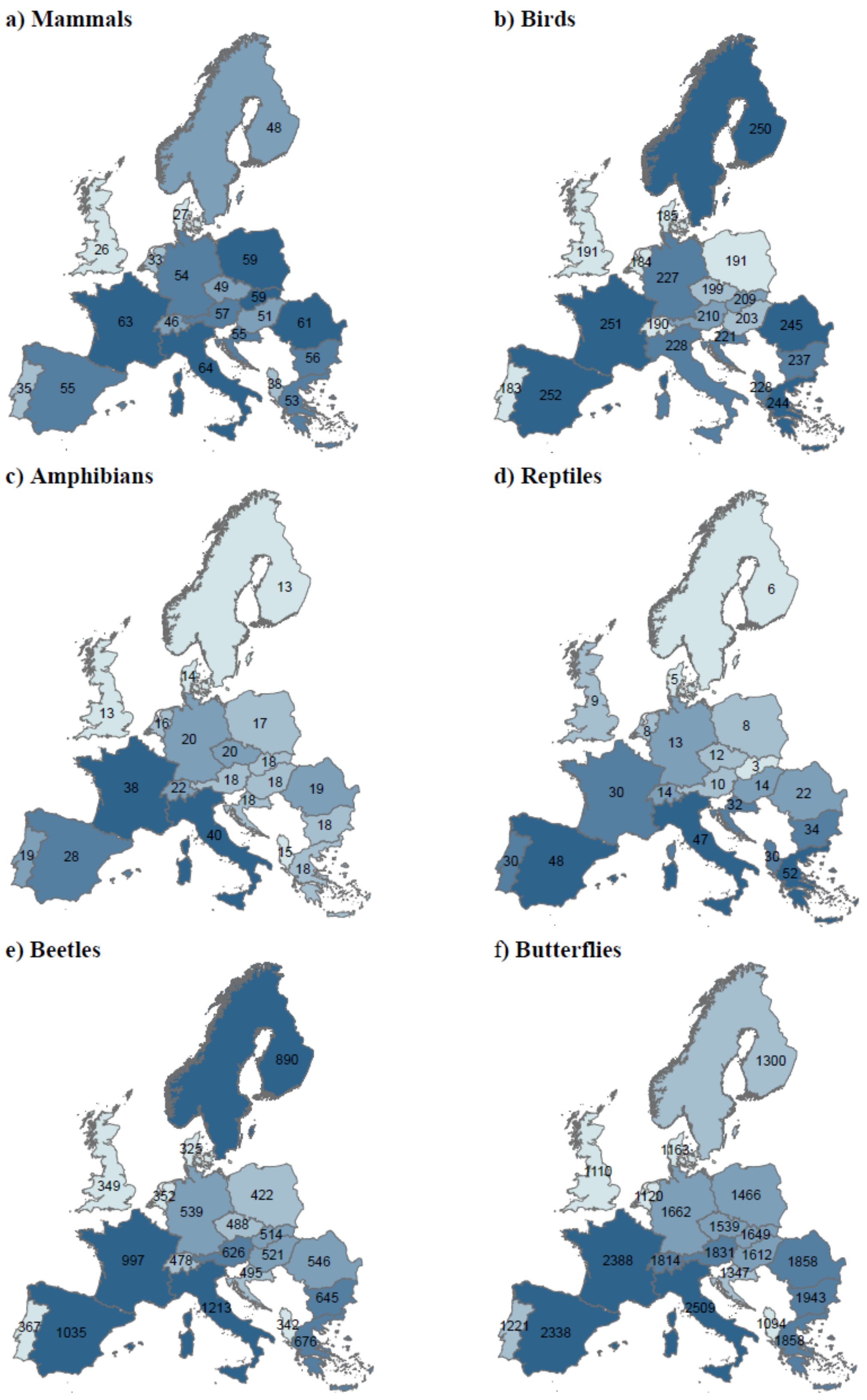

Supplement: Supplementary file 1 — Figure S1. Total species richness collected in 20 European regions analysed for testing the influence of surrogates of climate‐energy, habitat heterogeneity and vegetation diversity on six animal groups. [file ECE3-6-1515-s001.pdf]
